# *Chlamydia pneumoniae *induces aponecrosis in human aortic smooth muscle cells

**DOI:** 10.1186/1471-2180-5-2

**Published:** 2005-01-21

**Authors:** Claudia Dumrese, Christine F Maurus, Daniel Gygi, Mårten KJ Schneider, Michael Walch, Peter Groscurth, Urs Ziegler

**Affiliations:** 1 Division of Cell Biology, Institute of Anatomy, University Zürich, Switzerland; 2 Laboratory for Transplantation Immunology, Department of Internal Medicine, University Hospital Zürich, Switzerland

## Abstract

**Background:**

The intracellular bacterium *Chlamydia pneumoniae *is suspected to play a role in formation and progression of atherosclerosis. Many studies investigated cell death initiation versus inhibition by *Chlamydia pneumoniae *in established cell lines but nothing is known in primary human aortic smooth muscle cells, a cell type among others known to be involved in the formation of the atherosclerotic plaque. Type of cell death was analyzed by various methods in primary aortic smooth muscle cells after infection with *Chlamydia pneumoniae *to investigate a possible pathogenic link in atherosclerosis.

**Results:**

Chlamydiae were found to be localized up to 72 h post infection in aortic smooth muscle cells either as single bacteria or inside of large inclusions. Quantification of host cell death by lactate dehydrogenase release assay revealed strictly dose and time dependent lysis for all tested isolates of *Chlamydia pneumoniae*. Phosphatidylserine exposure was detected by flow cytometry in *Chlamydia pneumoniae *infected cells. Ultrastructure of *Chlamydia pneumoniae *infected human aortic smooth muscle cells showed extensive membrane- and organelle damage, chromatin condensation but no nuclear fragmentation. DNA fragmentation as well as cell membrane permeability was analyzed by TUNEL and NHS-biotin staining and occurred exclusively in cells carrying *Chlamydia pneumoniae *spots but not in smooth muscle cells with inclusions. These morphological features of cell death were not accompanied by an activation of caspase-3 as revealed by analysis of enzyme activity but involved mitochondrial membrane depolarization as shown by TMRE uptake and release of cytochrome c from mitochondria.

**Conclusion:**

This study provides evidence that *Chlamydia pneumoniae *induce a spot like infection in human aortic smooth muscle cells, which results in a chimeric cell death with both apoptotic and necrotic characteristics. This aponecrotic cell death may assist chronic inflammation in atherosclerotic blood vessels.

## Background

Infection with *Chlamydia pneumoniae *(Cpn) usually causes acute respiratory tract infections [1]. Chronical infection with Cpn may also contribute to formation and progression of atherosclerotic lesions apart from the classical risk factors such as hypertension, hypercholesterolemia and hyperlipidemia [2]. Cpn has been extensively studied in the context of atherosclerosis [3] because atherosclerosis and cardiovascular disease are the leading causes of death in the United States, Europe and much of Asia [4,5]. The obligate intracellular bacterium has been detected in atherosclerotic lesions by immunohistochemistry, polymerase chain reaction and electron microscopy [6-8] and has also been cultured from atheromatous plaques [9,10]. On the cellular level smooth muscle cells and macrophages in the intima have been found to be infected with Cpn [11,12]. In general terms, if the inflammatory response does not effectively neutralize or remove the offending agents, such as Cpn, it can continue indefinitely resulting in the progression of the disease [2].

*Chlamydiae *exhibit a unique developmental cycle with two morphological distinct infectious and reproductive forms: the elementary- and the reticulate body. The life cycle proceeds for 48 – 72 h and ends with pathogen release that may damage the host cells [13]. Infection with this pathogen is accompanied by cytoplasmic alterations and damage of the host cells [12]. A balance between pro- and anti-apoptotic influences by *Chlamydia *can be postulated. On one hand it has been shown that *Chlamydia psittaci *and *Chlamydia trachomatis *can induce apoptosis *in vitro *[14,15]. On the other hand established cell lines infected with Cpn or *Chlamydia trachomatis *were protected from apoptotic cell death induced by various stimuli [16,17]. Most of the studies investigating pro- and anti-apoptotic activity of Cpn were performed in tumor cells or established cell lines. Since the character of cell death is affected by host cell type and *Chlamydia *strain it is of relevance to determine cell death in *Chlamydia *infected primary cells [18]. As regards the role of Cpn in atherosclerosis we need to understand death promoting and inhibiting capacities of Cpn in primary cultures of vascular cells.

Human aortic smooth muscle cells (HASMC) play an important role in the development of atherosclerotic lesions [19]. Therefore, we sought to clarify the nature of cell death induced by Cpn in HASMC.

Apoptosis and necrosis represent two extreme morphologically defined forms of cell death [20-23]. Recently the hybrid term aponecrosis was introduced describing the incomplete execution of the internal apoptotic pathway and the following necrotic degeneration [24]. Whether smooth muscle cell death is apoptotic, necrotic or even aponecrotic in nature would predictably influence the inflammatory response in the plaque. Necrotic cells releasing their contents provoke inflammation, whereas in apoptosis dead cells are ingested by neighboring cells and, therefore, do not provoke an inflammatory response [25]. In order to characterize the fate of HASMC following infection with Cpn we analyzed cell death by morphological and biochemical methods.

## Results

### Various morphological characteristics of bacterial infection in HASMC

**Figure 1:**
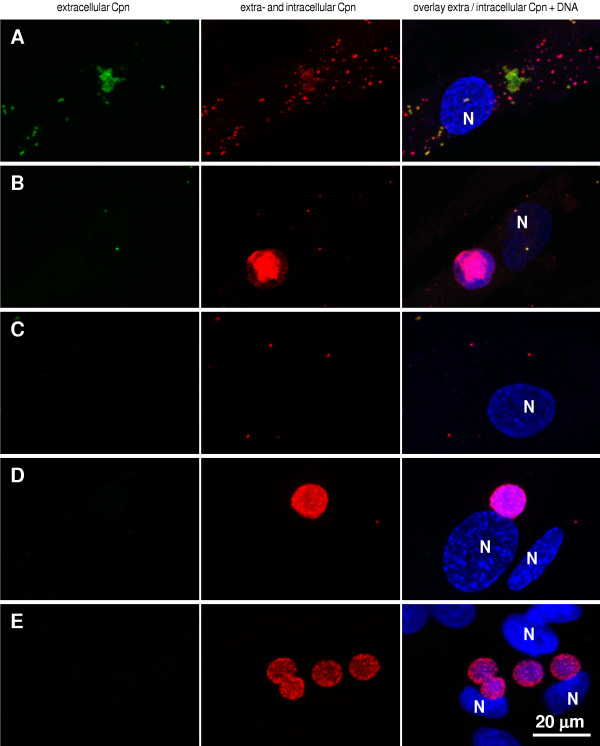
**Diverse morphology of Cpn infection. **HASMC (A, B, C and D) and HEp-2 cells (E) were infected for 72 h with Cpn-K6 at 128 IFU/cell (HASMC; A, B), Cpn-VR1310 at 8 IFU/cell (HASMC; C, D) and Cpn-K6 at 5 IFU/cell (HEp-2; E), respectively. Bacteria localized on the cell surface are stained with FITC (green), *Chlamydia *internally localized are stained with Texas Red (red) and FITC (for detail see material and methods). DNA of bacteria and host cells is stained with DAPI (blue). N = nucleus. Left row: extracellular Cpn. Middle row: extra- and intracellular Cpn. Right row: overlay of left- and middle row and DAPI staining. A, C: spot like infection of a HASMC; note that the majority of bacteria are localized intracellularly B, D: HASMC with a Cpn inclusion. E: Hep-2 cells with Cpn inclusions.

**Figure 2:**
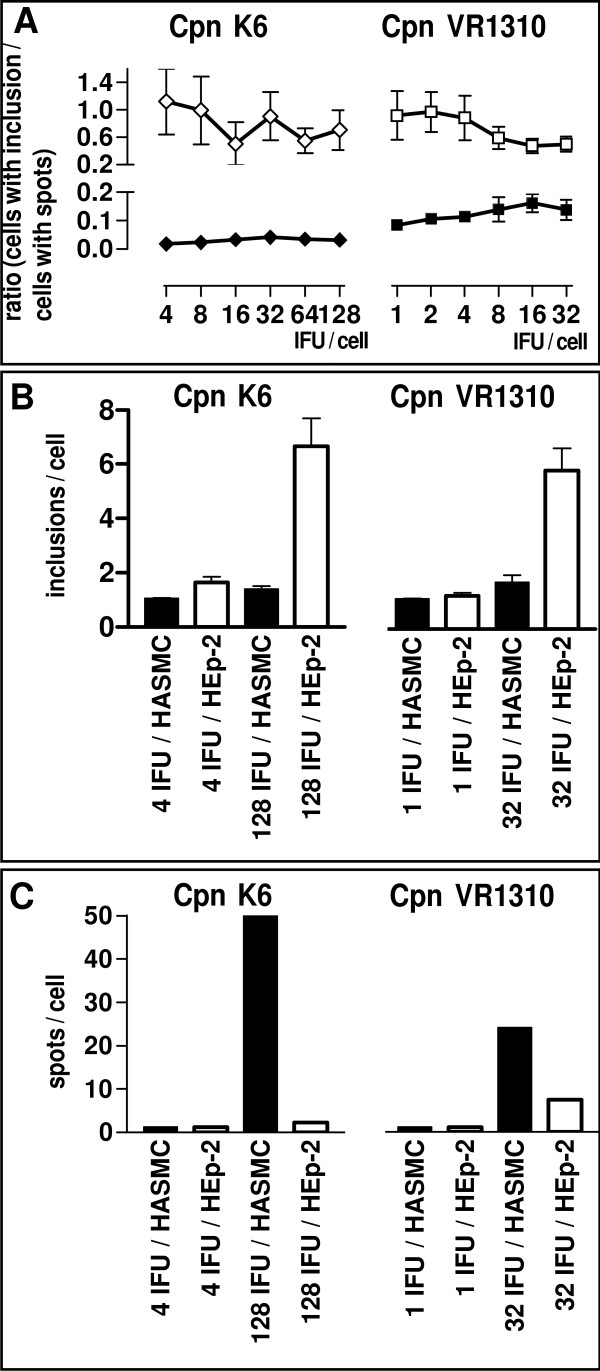
**Ratio of cells with inclusions versus cells with spots **HASMC and HEp-2 were infected with Cpn-K6 or Cpn-VR1310. Cells carrying inclusions or spots, respectively, were counted 48 h post infection. A: Depicted is the ratio of cells with inclusions versus cells with spots; -◆-: HASMC Cpn-K6 infected, -■-: HASMC Cpn-VR1310 infected, -◇-: HEp-2 Cpn-K6 infected, -□-: HEp-2 Cpn VR1310 infected B: inclusions / cell, C: spots / cell; black bars: HASMC, open bars: HEp-2 cells. The number of inclusions / cell in HASMC remained constant in contrast to the number of spots / cell when increasing the infectious dose, whereas in HEp-2 cells the number of inclusions / cell increases and the spots / cell remain constant.

In order to examine the morphological characteristics of Cpn infection HASMC as well as the tumor cell line HEp-2 were infected with Cpn-K6 and Cpn-VR1310, respectively, and stained after 24 h, 48 h and 72 h with anti-Cpn-MOMP antibody. According to the staining protocol intra- and extracellular localization of Cpn could be distinguished (Figure 1). Already after 24 h, but also after 48 h and 72 h, HASMC as well as HEp-2 cells were infected with numerous bacteria. Round or oval shaped smooth bordered intracellular inclusions were found in tumor cells (Figure 1E) as well as HASMC (Figure 1B,D). In contrast to tumor cells in primary HASMC Cpn were found also as single or rarely as irregular shaped, aggregated immunoreactive spots (Figure 1A, C, 4D). Quantification of morphology in HASMC revealed that after 72 h all cells were infected with at least one chlamydial spot (100% infection rate). Approximately 25% of the cells carried one or multiple inclusions. The different morphological characteristics in HASMC (inclusions, single and aggregated spots) could also be discriminated 48 h post infection. The ratio of cells carrying inclusions versus cells carrying spots was much higher in HEp-2 cells compared to HASMC irrespective of the infectious dose (Figure 2A). This ratio did not considerably vary between different Cpn strains K6 and VR1310 or as a function of the number of bacteria used for infection. Spots were usually much lower in number and not as prominently observed in HEp-2 cells compared to HASMC (Figure 2C). In HASMC the number of spots increased with higher infectious doses while at the same time the number of inclusions remained constant (Figure 2B) in contrast to Cpn infections of HEp-2 cells where more inclusions were formed at higher infectious doses without an increase of spots.

### Cpn lyse HASMC in a dose-dependent manner

To compare the lytic capacity of different Cpn isolates and to identify a possible time course of host cell death during the chlamydial infectious cycle, LDH release of HASMC was analyzed at 24, 48 and 72 h post infection.

**Figure 3:**
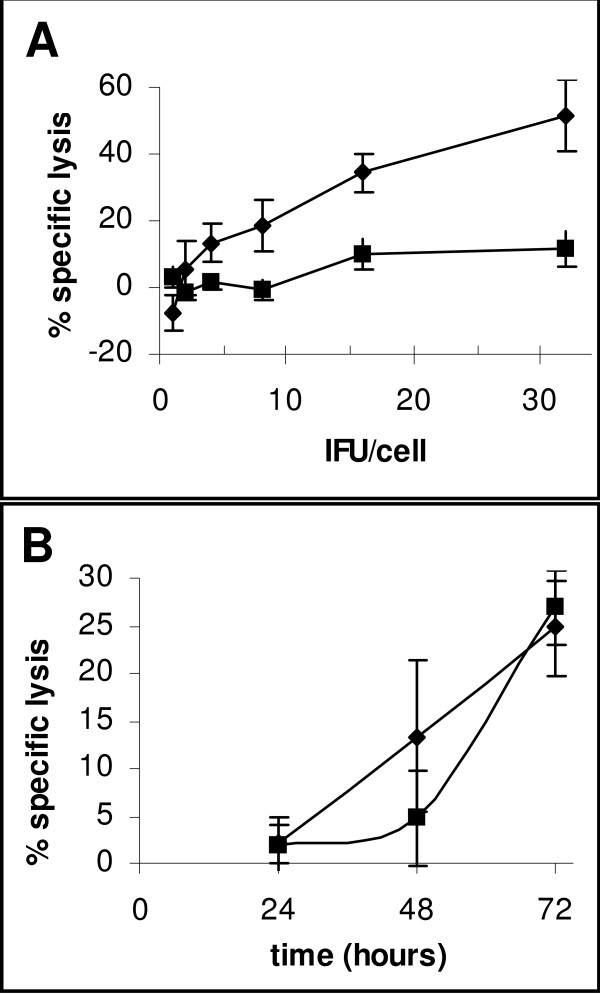
**Lytic capability of different Cpn isolates. **HASMC were infected with two different Cpn isolates. LDH release was measured in triplicates 24 h, 48 h and 72 h post infection and Cpn specific lysis was calculated. The average and standard deviation is plotted. One representative result out of three independent experiments is shown. A: Dose dependent Cpn specific lysis after 48 h revealed different capacities of the isolates tested to lyse HASMC. B: Different amounts of the various Cpn isolates leading to a comparable lysis over time of HASMC. Isolates were used as follows: Cpn-VR1310, 4 IFU/cell -◆-, Cpn-K6, 32 IFU/cell -■-

HASMC were inoculated with two different Cpn isolates: Cpn-VR1310 and Cpn-K6 at serial 2-fold dilutions ranging from 1 to 32 inclusion forming unit (IFU)/cell (Figure 3). Both isolates were found to be free of *Mycoplasma spec*. contamination as assessed by PCR. LDH release of mock treated HASMC or of cells inoculated with heat inactivated bacteria was comparable to the spontaneous LDH release of uninfected cells (data not shown).

Both Cpn isolates lysed HASMC in a strictly dose dependent fashion at all time points analyzed. Cpn mediated host cell lysis at 48 h post infection is shown in figure 3A. Data obtained from 24 h and 72 h post infection were similar and consistent with the displayed data subset. The levels of HASMC lysis induced by infection with 8 IFU/cell of Cpn-K6 (0.9% ± 3) and Cpn-VR1310 (18.6% ± 8) indicate large differences in the lytic capabilities of both Cpn isolates. Comparison of Cpn-induced LDH release at different time points (24, 48 and 72 h) demonstrated a constant increase in *Chlamydia*-specific HASMC lysis indicating continuous triggering of cell death throughout the whole infectious cycle (Figure 3B). In order to reach the same amount of HASMC lysis (27% ± 2) 4 IFU/cell of Cpn-VR1310 and 32 IFU/cell of Cpn-K6 respectively were required. This suggests that for a given degree of HASMC lysis either high amounts of a lytically less active isolate or lower amounts of a lytically more active isolate are required.

### Cpn infection of HASMC causes nuclear chromatin condensation as well as extensive damage of organelles and cell membrane

In order to discriminate between apoptotic and necrotic cell death of infected HASMC ultrastructural analysis by transmission electron microscopy (TEM) was carried out. As a control apoptosis was induced by treatment with the well described kinase inhibitor staurosporin.

**Figure 4:**
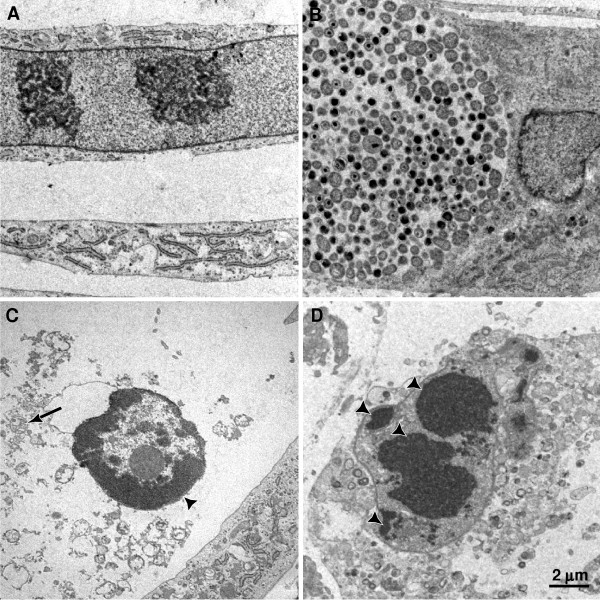
**Ultrastructure of Cpn infected HASMC. **Cells were either infected with 128 IFU/cell Cpn-K6 for 72 h (B,C) or incubated with staurosporin 1 μM for 16 h (D) or were left untreated (A). A: Untreated HASMC with normal ultrastructure, note the elongated nucleus with finely dispersed euchromatin. B: HASMC with a typical Cpn inclusion. The membrane bound vesicle contains dark (= elementary bodies) and pale (= reticular bodies) stained bacteria. The chromatin structure in the nucleus is unchanged. C: Cpn infected HASMC undergoing cell death. The nucleus is characterized by highly condensed heterochromatin (arrow head) and a compact nucleolus and is surrounded by dilate organelles (arrow). The cell membrane is completely disrupted. D: Staurosporin treated HASMC. The nucleus is partially fragmented containing patchy condensed heterochromatin (arrow heads).

Untreated HASMC had an elongated nucleus rich in euchromatin with one or two well-defined nucleoli (Figure 4A). The cytoplasm was scattered by numerous narrow-spaced profiles of rough endoplasmic reticulum (rER), a small Golgi complex and single elongated mitochondria. In Cpn infected cultures a variable number of HASMC harbored large inclusions containing many infectious elementary bodies and metabolically active reticular bodies (Figure 4B). The nucleus of these cells was slightly rounded but chromatin structure as well as morphology of organelles was unchanged. A large number of infected HASMC without Cpn inclusions were destroyed (Figure 4C). The round shaped nuclei showed a distinct condensation of heterochromatin and condensed nucleoli. Cell organelles including mitochondria and rER profiles were dilated and cell membranes were completely disrupted. Detection of single bacteria by TEM was possible only on rare occasions since distorted organelles in dying cells were barely distinguishable from Cpn.

Apoptotic HASMC treated with staurosporin showed a distinct condensation of chromatin (Figure 4D) and nuclei were fragmented frequently; damage of cell membranes and swelling of organelles appeared to a lower extent than in Cpn infected HASMC.

In general the overall ultrastructural morphology of HASMC infected with spot-like Cpn infection suggests a hybrid form of cell death: it shares chromatin condensation with apoptosis and damage of organelles and membranes with necrosis.

### Spot-like infection induces aponecrotic morphology in HASMC

The combination of chromatin staining by DAPI with labeling of DNA strand breaks via TUNEL staining and assessment of membrane integrity by NHS-biotin staining allows a distinction between necrosis as well as early and late phases of apoptosis using confocal laser scanning microscopy [23]. Cells dying by necrosis exhibit NHS-biotin labeling of the cytoplasm but not chromatin condensation or TUNEL staining of nuclei. Cells in early phases of apoptosis display nuclei with condensed or fragmented chromatin, with or without TUNEL staining, and are surrounded by NHS-biotin negative cytoplasm due to intact cell membranes. In late phases of the apoptotic cell death TUNEL positive nuclei occur together with a NHS-biotin positive cytoplasm.

**Figure 5:**
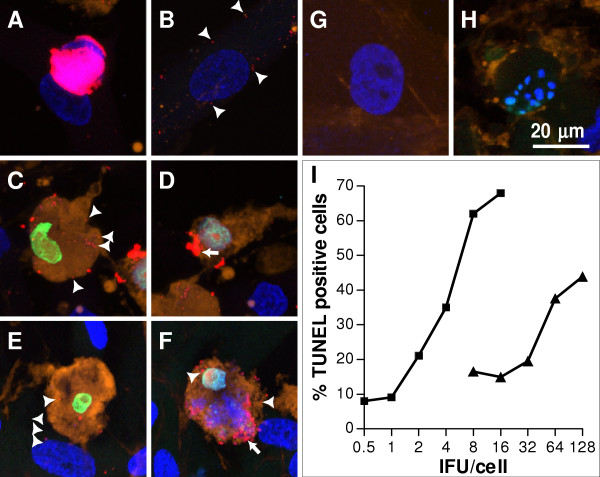
**Light microscopic analysis of the effect of Cpn infection on HASMC. **HASMC were infected with Cpn-K6 128 IFU/cell (A, B, C, D), Cpn-VR1310 32 IFU/cell (E, F) for 72 h or were inoculated with mock lysate (G) or treated with staurosporin 1 μM for 14 h (H). The cells were subsequently stained for TUNEL (green), NHS-biotin (brown), anti-Cpn-MOMP (red) and DNA (blue). A: HASMC bearing a Cpn-K6 inclusion. DAPI stained chromatin structure is normal and TUNEL as well as NHS-biotin labeling are negative. B: Cpn-K6 infected HASMC with multiple chlamydial spots (arrow heads) and normal nuclear structure. C, E: Cpn-K6 (C) or Cpn-VR1310 (E) infected HASMC with spot like infection (arrow heads). The condensed nucleus is TUNEL positive, the cytoplasm is diffusely labeled with NHS-biotin. D, F: HASMC with Cpn-K6 (D) or Cpn-VR1310 (F) in aggregated (arrow) spots and single spots (arrow heads). Note positive TUNEL labeling of the shrunken nucleus and NHS-biotin staining of the cytoplasm. G: Mock inoculated HASMC. Note the fine granular chromatin structure and lack of TUNEL- and cytoplasmic NHS-biotin staining. H: HASMC incubated with staurosporin. The nucleus exhibits a distinct nuclear fragmentation (DAPI staining) but no TUNEL labeling. The cytoplasm is NHS-biotin negative indicating an intact cell membrane. I: Quantification of TUNEL positive nuclei depending on the chlamydial infectious dose 72 h post infection reveals a dose dependent increase of TUNEL positive cells. Cells in 5 random fields with an area of 62'500 μm^2 ^were counted and the percentage of TUNEL positive cells was calculated. -▲-: Cpn-K6; -■-: Cpn-VR1310

HASMC were infected with Cpn for chosen periods of time or were inoculated with mock isolates or treated with staurosporin. Cells infected with Cpn-VR1310 (Figure 5E, F) showed an identical staining pattern as cells infected with Cpn-K6 (Figure 5A – D).

Mock inoculated cells displayed round or oval shaped nuclei with finely dispersed chromatin visualized by DAPI staining and were always TUNEL negative. NHS-biotin was bound exclusively to the cell surface indicating intact membranes (Figure 5G).

In Cpn infected cultures a variable labeling pattern of HASMC was observed. Cells with Cpn inclusions always displayed normal chromatin staining and no TUNEL or NHS-biotin labeling (Figure 5A). HASMC with spot-like infection either contained an unaltered nucleus and NHS-biotin negative cytoplasm (Figure 5B) or showed a TUNEL positive nucleus with condensed chromatin embedded in NHS-biotin stained cytoplasm (Figure 5C, E). In HASMC displaying both Cpn spots and aggregates the nucleus was always labeled by TUNEL and the cytoplasm by NHS-biotin (Figure 5E, F). Cpn-infected HASMC never showed membrane damage in combination with an unaltered nucleus, representing necrotic cell death. Cpn-infected HASMC displaying TUNEL positive nuclei and damaged membranes could be detected at 24, 48 and 72 h post infection. 8 h post infection was the earliest time point when TUNEL positive nuclei could be detected, suggesting induction of apoptosis during the whole life cycle of Cpn (not shown). TUNEL-positive HASMC were found even at infectious doses of 1 IFU / cell for Cpn-VR1310 and 8 IFU / cell for Cpn-K6 and the amount of TUNEL-positive HASMC increased strictly dependent on the chlamydial dose used for infection (Figure 5I). Again, both Cpn strains used in this study elicited the same features of cell death in HASMC according to the infection morphology. Identical to the lytic capacity (Figure 3A) of these strains, K6 had to be used at higher IFU compared to VR1310 to generate a similar number of cells with TUNEL-positive nuclei and NHS-biotin positive cytoplasm. In contrast to HASMC neither Cpn-K6 nor Cpn-VR1310 induced a concentration dependent increase in TUNEL positive nuclei in HEp-2 cells and numbers of TUNEL positive nuclei remained below 10% (not shown).

In staurosporin treated cells the rounded nuclei were fragmented or exhibited chromatin that was condensed marginally, whereas NHS-biotin labeling was predominantly negative (Figure 5H). Just a minority of the cells revealed a condensed TUNEL-positive nucleus and at the same time were also NHS-biotin positive reflecting late stages of apoptotic cell death.

### Cpn infection induces phosphatidylserine externalization in HASMC

**Figure 6:**
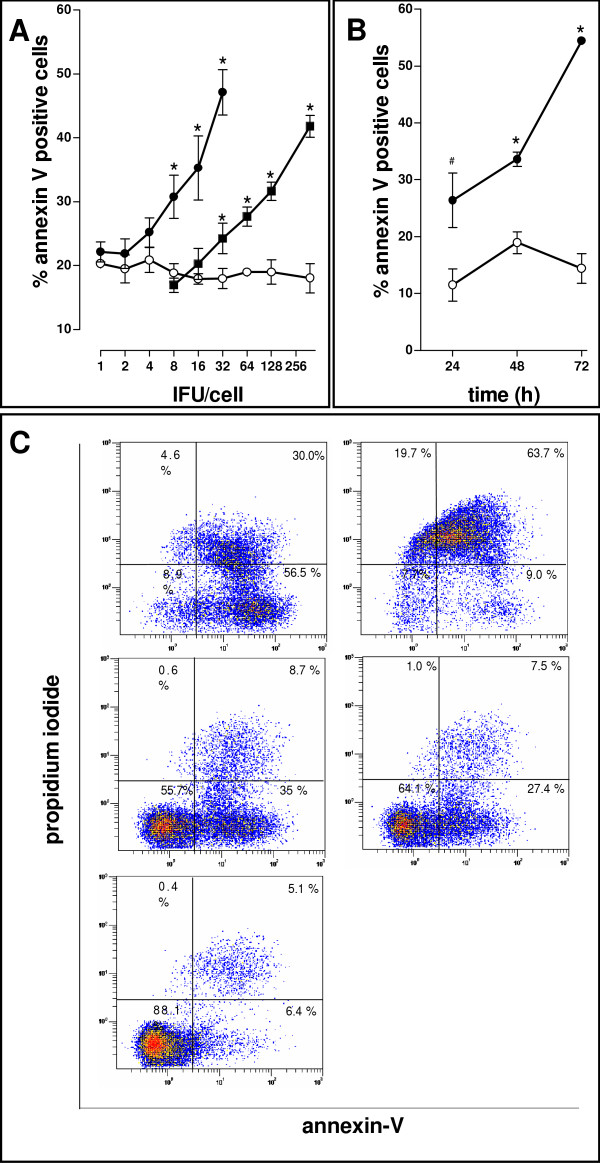
**Dose response relationship between bacterial titer and phosphatidylserine exposure in Cpn infected HASMC. **Cells infected with Cpn-K6 in doses between 8 – 386 IFU/cell or with Cpn-VR1310 in doses between 1 – 32 IFU/cell were stained with annexin-V / propidium iodide and analyzed by flow cytometry 48 h post infection. Mock inoculated cells are shown as controls. In figure A and B pooled data out of three independent experiments are displayed. A: The amount of single annexin-V positive cells increased dependent on the chlamydial infectious dose (-◆ -: Cpn-K6; -●-: Cpn-VR1310) but not in mock treated (-○-) cells. (*: significant compared to mock treated cells with p < 0.001) B: the number of single annexin-V positive cells increased over the time when cells were infected with Cpn-K6 128 IFU/cell (-●-) but not if they were mock treated (-○-) (significant compared to mock treated cells with p < 0.001)(#)). C: Dot plots of one representative experiment are depicted to illustrate the amount of annexin V / propidium iodide double labeled cells. Upper left panel: HASMC treated with staurosporin (apoptosis). Upper right panel: HASMC treated with Na-azide (necrosis). Middle left panel: HASMC infected with Cpn-VR1310 at 32 IFU / cell 48 h post infection. Middle right panel: HASMC infected with Cpn-K6 at 128 IFU / cell 48 h post infection. Lower left panel: mock treated HASMC.

HASMC were infected for 48 h with Cpn-K6 or -VR1310 in 2-fold dilutions ranging from 8 to 386 IFU/cell or from 1 to 32 IFU/cell, respectively. As controls cultures were inoculated with mock isolates or with heat inactivated bacteria (data not shown) and analyzed for annexin-V binding and propidium iodide uptake. Uninfected as well as Na-azide treated HASMC served as negative and staurosporin-treated cells as positive controls for apoptosis (Figure 6C upper right and upper left panel). Control cultures inoculated with mock lysate (Figure 6) or heat inactivated bacteria and untreated cells showed always less than 20% annexin-V single positive cells. This proportion of annexin-V positive cells represented background staining, probably due to the experimental procedure. Upon infection with 128 IFU/cell of Cpn-K6 for 72 h the number of single annexin-V positive cells increased to more than 50% (Figure 6B). Upon Cpn infection annexin-V and propidium iodide double-positive staining reached 10% as compared with 4% in mock-inoculated and 3% in untreated HASMC (not shown). In staurosporin treated cultures the amount of both annexin-V single and double positive cells increased strongly (Figure 6 upper left panel) indicating a continued induction of classical apoptotic cell death.

The amount of annexin-V single positive HASMC infected with Cpn increased linearly in a dose- (Figure 6A) and time- (Figure 6B) dependent manner. Cpn-VR1310 again induced phosphatidylserine externalization at much lower doses compared to Cpn-K6. Neither mock inoculation (Figure 5A, B6C lower left panel), nor inoculation with heat inactivated bacteria (data not shown), induced apoptosis. This indicates that infection with living bacteria is required for induction of cell-death.

Taken together, the dose- and time dependence between infection and phosphatidylserine externalization were consistent with induction of early apoptotic events.

### *Chlamydia *infection in HASMC induces caspase-3 independent cell death by dissipation of mitochondrial membrane potential

**Figure 7:**
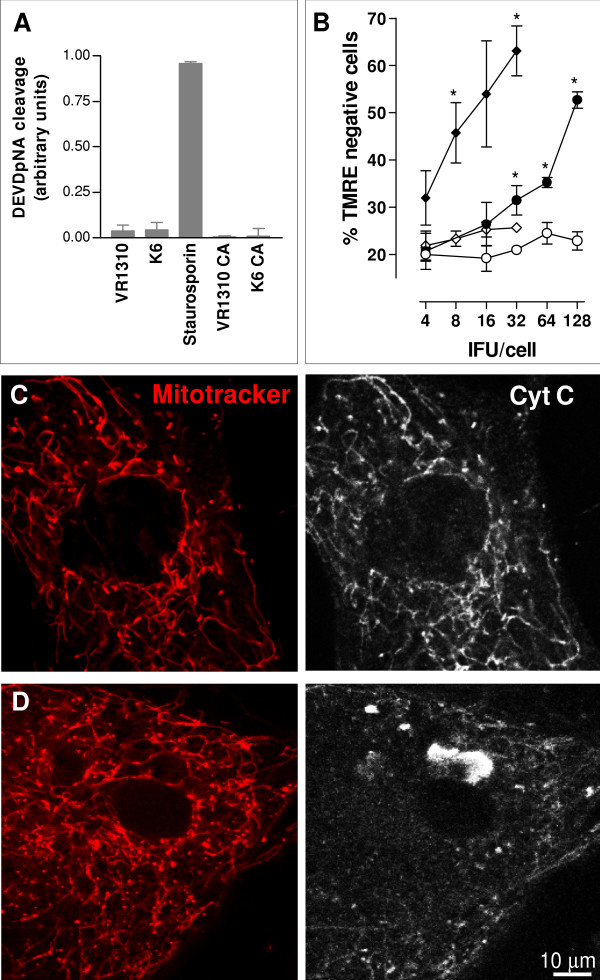
**Caspase 3 activity and mitochondrial membrane permeability **A: Caspase-3 activity was assessed in HASMC infected with 128 IFU/cell Cpn-K6 ± Chloramphenicol (CA), loaded with mock isolate or treated with 1 μM staurosporin for 14 h. No caspase-3 activity can be detected in Cpn infected or mock inoculated cells in contrast to staurosporin treated cells. B: HASMC were infected with 4 – 128 Cpn-K6 (-●-) or 4 – 32 IFU/cell Cpn-VR1310 (-◆-) and stained by TMRE for detection of mitochondrial membrane potential in combination with propidium iodide. Propidium iodide negative cells were analyzed by flow cytometry for TMRE staining. Infected HASMC treated with 0.1 μg/ml chloramphenicol served as controls (open symbols). Loss of TMRE staining is induced in HASMC infected with viable Cpn but not in cells infected with Cpn treated with chloramphenicol. Mean values with standard error of three independent experiments are shown. C, D: HASMC were infected with Cpn-K6 (IFU 128 IFU/cell, 48 h) and stained for cytochrome c (white). Mitochondria were labeled with mitotracker red (red). Loss of cytochrome c staining is found in mitochondria of HASMC infected with Cpn (D) but not in mock treated cells (C).

After induction of apoptosis an enzymatic cascade is activated leading to the disassembly of the cell [23]. Key enzyme in this cascade is caspase-3. However, in some cases apoptosis can proceed without involvement of caspase-3 [26]. In order to investigate the role of caspase-3 HASMC were infected with Cpn-K6 128 IFU/cell or Cpn-VR1310 32 IFU/cell, respectively, for 48 h (24 h and 72 h not shown) or treated with 1 μM staurosporin for 12 h to induce apoptosis. Samples were analyzed for caspase-3 activation by measuring enzyme activity after lysing the cells. No activity of caspase-3 in Cpn infected HASMC (Figure 7A) could be detected. In contrast distinct caspase-3 activity was found in staurosporin-treated cells.

A major role in the induction and regulation of cell death is played by mitochondria. Pro-apoptotic factors result at mitochondria in the dissipation of the mitochondrial membrane potential and release of cytochrome c. After infection of HASMC with Cpn-K6 or Cpn-VR1310 cells were loaded with TMRE (tetramethylrhodamineethylester) [27], a dye, that is only taken up into mitochondria with an intact mitochondrial potential [28]. Cells with intact membranes excluding propidium iodide were analyzed by flow cytometry.

The number of TMRE negative cells strictly increased with the chlamydial dose whereas blocking of chlamydial protein synthesis by addition of chloramphenicol completely abrogated this effect (Figure 7B). Moreover, labeling of Cpn-K6 infected or mock treated cells for cytochrome c and mitotracker red revealed distinct loss of cytochrome c staining of the mitochondria in the infected population (Figure 7D) but not in mock treated cells (Figure 7C).

Taken together, the data suggest *Chlamydia pneumoniae *induce caspase-independent apoptosis-like cell death in infected HASMC by involving mitochondrial membrane dissipation resulting in the release of cytochrome c.

## Discussion

In our in vitro study cell death was only induced in HASMC by infection with viable Cpn but not after incubation with heat inactivated or chloramphenicol treated bacteria. Additionally, different Cpn isolates showed various lytic activities towards host cells that correlated to different reproduction rates in tumor cells as measured by real time PCR [29]. A reproductive infection would seem to be a prerequisite for cell death induction in HASMC. The observation that heat inactivated or chloramphenicol treated bacteria never caused changes of the host cells underlines that cell death induction is not a bystander effect of a potential cytopathic chlamydial component such as the heat stable LPS. It can be concluded that HASMC death depends on internalization of the *Chlamydia *and is associated with the suppression of bacterial replication and productive infection. The necessity for a reproductive infection was also shown for *Chlamydia psittaci *which induced caspase 3 independent apoptosis in macrophages and epithelial cells only after reproductive infection along with bacterial protein synthesis [15]. Cell death induction was never found in cells harbouring inclusions which normally are viewed as the manifestation of a reproductive infection. HASMC formed inclusions with a relatively low frequency compared to tumor cells. In contrast, spots resulting presumably from single bacteria were found frequently illustrating a different infection morphology of Cpn in these primary vascular cells. Currently no data are available regarding metabolism, protein synthesis and reproduction or functional relation with host cells of these single, spot-like bacteria. From our data it only can be concluded that these single bacteria are sensitive to chloramphenicol which inhibits protein synthesis in Cpn and abrogates cell death induction. Cell death seems to result from an interaction of the host cell with these single bacteria as it is ongoing for extended times.

Cpn induced cell death in HASMC did not fulfill all criteria for apoptosis or classical necrosis. Condensation of chromatin, positive TUNEL staining, externalization of phosphatidylserine, inhibition of TMRE labeling and loss of cytochrome c immunoreactivity in mitochondria were clear markers for apoptosis. But early damage of cell membrane and organelles as found by TEM and NHS-biotin labeling indicated necrotic type of cell death in Cpn infected cells. According to previous classifications Cpn mediated cell death belongs to apoptosis-like cell death [30] or can be termed aponecrosis due to characteristic ultrastructural features [24]. Cpn induced cell death in HASMC having features of necrosis and apoptosis is in agreement with a study employing mouse embryonic fibroblasts infected with *C. trachomatis *which died through a combination of necrosis and apoptosis [18].

If cell death induction by Cpn in HASMC is caspase-3 independent the question arises about alternative pathways that are involved. Mitochondria are important regulators of cell death and loss of mitochondrial permeability transition is an important indication for apoptosis [31]. Here we show by TMRE labeling dissipation of the mitochondrial potential of *Chlamydia *infected cells. This effect could be inhibited by blocking bacterial protein synthesis using chloramphenicol. Moreover, cytochrome c was released from mitochondria of infected HASMC as indicated by loss of immunorecactivity. The important role of mitochondria in *Chlamydia *induced cell death has also been shown in tumor cell lines infected with *Chlamydia psittaci *[14]. Pro-apoptotic Bcl-2 family members like Bax were activated and apoptosis induction occurred during the whole chlamydial life cycle. Apoptosis induced by *Chlamydia psittaci *was blocked in cells over expressing Bax inhibitor and anti-apoptotic Bcl-2 [14]. Bax^-/- ^mouse embryonic fibroblasts were shown to be resistant to *Chlamydia muridarum*-induced apoptosis and fewer bacteria were recovered after two infection cycles. This suggests that Bax-dependent apoptosis may be used to initiate a new round of infection [32]. In a second study, *Chlamydia trachomatis *infected mouse fibroblasts showed a higher level of apoptosis than *Chlamydia *infected HeLa cells as measured by annexin V / propidium iodide double labeling [18]. This not only shows that Bax activation could reflect a stress response in infected cells [33] but that primary cells show a different stress response to *Chlamydia *infection than tumor cells.

Beside Bax other factors like apoptosis-inducing factor (AIF) may be involved in caspase-independent cell death [34] but they have never been investigated in *Chlamydia*-induced cell death. AIF which is upregulated and translocated from the mitochondria to the cytosol may play an important role in induction of caspase-3 independent cell death [34-36]. Injection of AIF into the cytoplasm induces similar chromatin condensation coupled to phosphatidylserine exposure as we found in Cpn infected HASMC [37]. Future experiments will have to show if AIF, Bax or other Bcl-2 family members are involved in Cpn induced HASMC death.

There exist numerous studies showing that Cpn and *Chlamydia trachomatis *prevent cells in vitro from undergoing apoptosis [16,17,38-44]. However, these experiments were performed on established cell lines. Our study shows that in primary cultures of cells such as HASMC various types of Cpn infection occur. Spot like infection resulted in aponecrosis whereas cells with Cpn inclusions appeared to be prevented from cell death. Apparently Cpn in inclusions have evolved strategies to inhibit cell death presumably to complete the developmental cycle. About possible mechanisms can be speculated: it has been shown that Cpn inserts components of a type-III secretion system into the inclusion membrane [45] and releases factors into the host cell [46-48] that affect host cell metabolism. Cpn secretes proteins that translocate from the inclusion to the cytoplasm [47,49]. CADD, a secreted protein from *Chlamydia trachomatis*, has been shown to interact with death domains and co-localizes with Fas. Recombinant CADD has been shown to induce caspase dependent apoptosis in several tumor cell lines [50]. Therefore, other yet unidentified proteins from *Chlamydia *could interact with the host cell death machinery and inhibit or promote cell death.

Currently, it is unclear how the observed morphology of Cpn in HASMC and the induced aponecrotic cell death affects the progression of atherosclerosis. Cpn infection of HASMC was accompanied by phosphatidylserine externalization which might result in the ingestion of dying cells by macrophages. Macrophages will then either stop further progress of Cpn mediated damage or in turn become activated and thereby contribute to inflammation in the atherosclerotic vessel [51,52]. Early membrane damage found in the aponecrotic HASMC may provoke or assist inflammation in the atherosclerotic vessel due to release of cellular constituents. Finally damage of vascular smooth muscle cells by Cpn infection might lead to plaque instability [19].

## Conclusions

Cpn infection of cultured HASMC results in cell death that can be termed aponecrosis due to the sharing of apoptotic and necrotic features. This form of cell death occurs just in cells bearing single or aggregated bacteria but not in cells with inclusions. Aponecrotic HASMC may in vivo assist chronic infection in the vascular wall supporting the progression of atherosclerosis.

## Methods

### Chlamydial organisms, cells, antibodies and reagents

Cpn-VR1310 and Cpn isolate Kajaani 6 (Cpn-K6) were kindly provided by G. Christiansen (Institute of Microbiology and Immunology, University Aarhus, Denmark) and M. Puolakkainen (Haartman Institute, University of Helsinki, Finland), respectively.

HEp-2 cells obtained from ATCC (Manassas, USA) were cultivated in MEM-Eagle's (Gibco BRL, Invitrogen, Basel, Switzerland) and HASMC purchased from Clonetics (Verviers, Belgium) were maintained in DMEM supplemented with 10% FCS, 2 mM glutamine, all from Gibco BRL, Invitrogen, and 10 μg/ml neomycin (Sigma Chemicals, Buchs Switzerland). Cells and chlamydial organisms were tested for *Mycoplasma spec*. contamination by PCR [53] and 4',6-Diamidin-2'-phenylindol-dihydrochloride (DAPI) (Roche, Mannheim, Germany) staining and were mycoplasma free.

Anti-Cpn-MOMP monoclonal antibody was from DAKO (Zug, Switzerland); anti-cytochrome c antibody raised in sheep was from Sigma, FITC-labeled monoclonal anti-*Chlamydia*-LPS antibody from Biorad (Redmont, USA). Donkey anti-mouse polyclonal antibody was obtained from Jackson Immuno Research (Baltimore, USA) and staurosporin from Sigma Chemicals.

### Chlamydial culture and infection of HASMC

Cpn was cultured according to an established protocol [54]. Briefly, confluent HEp-2 cells were inoculated with Cpn at 1 IFU/cell in medium, centrifuged at 1000 × g for 1 h and grown for 72 h in medium containing 10% FCS, 2 mM glutamine, 10 μg/ml neomycin and 2 μg/ml cycloheximide (Sigma). Subsequently, cells were harvested, disrupted by sonification and bacteria were purified on a discontinuous renografin (Sigma Chemicals) gradient as previously described [55]. Non infected HEp-2 cells were subjected to the same harvesting / purification procedure and referred to as mock controls. In order to determine the chlamydial titer (IFU/ml), confluent HEp-2 cells grown on cover slips were infected with serial 2-fold dilutions of purified bacteria. After 72 h cells were fixed, stained with anti-*Chlamydia*-LPS antibody, inclusions were counted under a fluorescence microscope and the titer was calculated.

HASMC were seeded into 6-well plates (Nunc, Roskilde, Denmark) at a density of 2.4 × 10^4 ^cells/cm^2 ^one day prior to infection. Infection with chlamydial organisms was performed in DMEM using titers between 8 and 386 IFU/cell which were added to HASMC, centrifuged onto the cells for 1 h at 1000 × g. Subsequently, the supernatant was replaced by DMEM containing 10% FCS, 2 mM glutamine and 10 μg/ml neomycin. In some experiments chloramphenicol 0.1 mg/ml was added analysis or cells were treated with 1 μM staurosporin for 14 h. Cells were grown at 37°C in the presence of 5% CO_2 _until they were analyzed.

### Determination of infection morphology, intra- versus extracellular staining of Cpn

Live HASMC and HEp-2 cells were incubated with anti-Cpn-MOMP antibody (1:50) in PBS + 2% BSA for 1 h at room temperature (RT). After washing and fixation with 2% paraformaldehyde + 3% sucrose in PBS at RT, cells were incubated for 1 h with donkey anti-mouse-FITC antibody, diluted 1:100 in PBS + 2% BSA. Bacteria located extracellularly are stained exclusively green by this step. Subsequently, cells were lysed with 0.1% Triton-X 100 for 1 min at RT, rinsed with PBS + 2% BSA for 20 min, and incubated with anti-Cpn-MOMP antibody (1:50) followed by Texas Red-labeled donkey anti-mouse antibody (1:200) in 2% BSA in PBS. The second staining step identifies both intracellular and extracellular bacteria.

Counting of cells with inclusions or spots as well as the number of inclusions and spots were performed on volume data obtained using a laser scanning microscope (SP2, Leica, Heidelberg, Germany) followed by image analysis using the software package Imaris (Bitplane, Zurich, Switzerland).

### Determination of cytotoxicity by lactate dehydrogenase release (LDH)

LDH release of HASMC was assessed using a cytotoxicity detection kit (Roche) following the manufacturer's instruction. In brief, HASMC were seeded in a 96-well plate (NUNC) at a density of 2.4 × 10^4 ^cells/cm^2^, infected with Cpn between 2 and 32 IFU/cell and cultivated for 24 h, 48 h and 72 h. For cell death analysis 100 μl of cell-free supernatant was harvested, mixed with dye solution, incubated for 20 min and absorption was measured at 490 nm. Percent *Chlamydia*-specific lysis was calculated according to the formula: ((experimental value – spontaneous release)/(maximum release – spontaneous release) × 100. Spontaneous release corresponded to untreated HASMC and infected cells lysed additionally with Triton-X 100 showed the maximal release.

### Transmission electron microscopy (TEM)

Cells were fixed in 50 mM sodium cacodylate buffer pH 7.3 containing 2% glutaraldehyde and 0.8% paraformaldehyde and postfixed with 1% OsO_4 _in 50 mM sodium cacodylate buffer, pH 7.3 dehydrated in an ethanol series and embedded into epon (Catalys). Ultrathin sections of 50 nm were contrasted with uranyl acetate and lead citrate and studied with a CM 100 transmission electron microscope (Phillips, Leiden, Netherlands).

### TUNEL-, NHS-biotin-, Cpn- and DAPI-staining for analysis by confocal microscopy

Cells were trypsinized, washed in PBS and incubated with 0.1 mg/ml NHS-biotin (Pierce, Rockford, USA) in PBS for 30 min on ice, fixed with 3% paraformaldehyde + 2% sucrose in PBS and cytospinned onto glass slides. Samples were permeabilized with 0.1% Triton-X 100 in PBS for 1 min at RT and stained for DNA strand breaks using terminal transferase (Roche), incorporating dUTP-FITC (Roche) overnight at 37°C as previously described [56]. Subsequently, cells were incubated with anti-Cpn-MOMP antibody (1:50) and detected with donkey anti-mouse Texas Red antibody (1:200) in 2% BSA in PBS for 1 h, followed for 45 min by 0.5 μg/ml streptavidin-Cy5 (Jackson Immuno Research) detection of NHS-biotin. Nuclei and DNA of bacteria were stained with 1 μg/ml DAPI in PBS for 10 min. Samples were then embedded in fluorescence mounting medium (Dako) and analyzed using a confocal laser scanning microscope (SP2, Leica, Heidelberg, Germany).

### Annexin-V/propidium iodide staining for flow cytometry

Phosphatidylserine exposure and propidium iodide incorporation was assessed using the annexin-V-FLUOS staining kit (Roche). In brief, supernatants of HASMC cultures were collected and mixed with trypsinized cells. Samples were washed in PBS followed by 20 min staining with annexin-V / propidium iodide staining solution and analyzed by flow cytometry (Becton Dickinson, Basel, Switzerland).

### Determination of caspase-3 activity

0.2 × 10^6 ^HASMC consisting of floating and adherent cells were lysed at 4°C in cell lysis buffer (20 mM PIPES, pH 7.2, 100 mM NaCl, 1 mM EDTA, 10 mM DTT, 0.1% CHAPS, 10% sucrose), subjected to three freeze-thaw cycles and centrifuged at 14000 × g for 10 min. Protein content of supernatants was measured using the micro BCA protein assay reagent kit (Pierce). Cell lysates containing 40 μg protein were incubated with DEVD-*p*-nitroanilide (0.8 mM) in lysis buffer and absorption was measured every 5 min in a spectrophotometer at 405 nm for 6 h at 37°C. Caspase-3 activity was calculated as the initial ascending slope of absorption depicted as arbitrary units.

### TMRE and propidium iodide staining for analysis of mitochondrial membrane potential

Cells were stained with TMRE 100 nM and propidium iodide 2 μg/ml in DMEM with 10% FCS for 30 min at 37°C and 5% CO_2_, collected by trypsinization, combined with detached cells at time of trypsinization and analysed by flow cytometry (Becton Dickinson).

### Cytochrome c and mitotracker red staining for confocal microscopy analysis

Cells were incubated in the presence of 50 nM mitotracker red for 30 min, subsequently fixed with 3% paraformaldehyde + 2% sucrose and were cytospinned onto glass slides. Samples were permeabilized with 0.1% Triton-X 100 in PBS for 1 min at RT. Subsequently stained with sheep anti cytochrome c (1 : 2000) in 3 % BSA in PBS for 1 h at 37°C followed by detetion with donkey anti sheep biotin antibody (1:200) in 0.5 % BSA in PBS for 1 h at RT. Cells were labeled with streptavidin Cy-5 (1 : 200) for 1 h at RT and analysed under a confocal laser scanning microscope.

## Authors' contributions

CD performed all infections and analyzed morphological markers for cell death.

CFM and MKJS performed FACS experiments.

DG initiated the study and performed the first infections of vascular cells.

MW analyzed viability experiments and provided input for exeriments.

PG provided critical intellectual input to the study and organized financial support.

UZ established assays for the detection of caspase 3, morphological cell death markers and was leading the study.
